# Editorial for the Topic on Microdevices for Biomedical Analysis

**DOI:** 10.3390/mi13040570

**Published:** 2022-04-03

**Authors:** Kosuke Ino

**Affiliations:** Graduate School of Engineering, Tohoku University 6-6-11, Aramaki-aza Aoba, Aoba-ku, Sendai 980-8579, Japan; kosuke.ino@tohoku.ac.jp

Recently, biomedical tools have been rapidly miniaturized due to the progress of micro-/nanofabrication technology based on bottom-up and top-down approaches. Due to miniaturization, the size and weight of biomedical tools has considerably decreased, leading to lower power consumption, higher portability, lower cost, higher precision, and quicker analysis. In addition to miniaturization, microdevices can exhibit special phenomena in micro-/nanospaces and/or micro-/nanostructures. For example, microfluidic devices can easily induce laminar flow, which is useful for biomedical applications, such as cell culture and bioanalysis. This editorial summarizes papers on microdevices for biomedical analysis published in 2020 in *Micromachines*.

Microdevices have been used for bioapplications of retinal tissues. Ha et al. [1] reported microelectrode array (MEA) devices for in vitro and ex vivo electrophysiological analyses using animal retinal tissues. In conventional devices, an MEA is prepared on a plane, leading to the deformation of hemispherical retinal tissues on the planar MEA. The deformation is unsuitable for these assays. In contrast, their devices consist of a hemispherical MEA, which allows retinal tissue to adhere closely to electrodes without tissue deformation. In this study, a polydimethylsiloxane (PDMS) layer with wavy Pt electrodes was stretched and placed on a hemispherical layer. The devices successfully recorded spontaneous neural activity from the retinas of mice without the deformation of the retina. The developed fabrication techniques will be applied to wearable sensors for health monitoring. Shire et al. [2] reported a high-density subretinal visual prosthesis, specifically, an implantable device consisting of a flexible electrode array and layer-by-layer fabrication schemes for multilayered electrode arrays.

Microdevices have been widely utilized in cell culture platforms for microphysiological systems, including organs on a chip. For example, skin models have been widely investigated because the physiological and immunological barriers of the skin prevent infection. Jahanshahi et al. [3] reported a skin model consisting of a multilayer epidermis on a hydrogel scaffold and a three-dimensional vascular-like network. To prepare for the vascular-like network, sacrificial Pluronic F127 inks were printed in a gelatin/transglutaminase solution. Compared with the monolayer of cells on gelatin, the electrical resistance of the cellular structure was dramatically enhanced. In addition, the model was used to investigate the inflammatory response of keratinocytes following an *Escherichia coli* infection. In this study, the expression levels of proinflammatory cytokines were measured. The authors reported that this model has great potential for drug testing.

Microdevices have also been used for the investigation of mechanical stimulation of cellular responses. Yadav et al. [4] reported the effects of mechanical stimulation on Rho protein markers (RhoA and Rac1) in liver cancer cells. The Rho family regulates intracellular actin dynamics. In this study, liver cancer cells were seeded onto a membrane that was placed and stretched using an electromagnetic actuation device. After stimulation, the cells were lysed, and the protein expression level was quantified by enzyme-linked immunosorbent assay. In this study, RhoA and Rac1 were overexpressed after stretching. The authors suggest that these markers can also be used as diagnostic biomarkers for liver cancer cells.

Cell-based biosensing has also been reported. Lee et al. [5] reported sensors for high-throughput assessment of bisphenol A (BPA) detoxification using micropillar/microwell chips. A droplet of alginate solution containing liver cells was placed on the micropillar of the chip. After alginate gelation, the micropillars were immersed in the microwell chip filled with culture medium containing analytes. The microdevice evaluated potential BPA detoxification using Korean pear extract. In this study, BPA toxicity was reduced by using Korean pear extract.

Luo et al. [6] developed an integrated microdevice for the continuous perfusion and temperature control in microenvironments for the culturing and monitoring of coral polyps. To mimic the marine ecological environment, a microwell-based microfluidic platform was fabricated and a low shear rate and high substance transfer were achieved. In this study, individual coral polyps were monitored during temperature changes. According to this study, coral polyps gradually lost their biological activity at high temperatures.

Microdevices have also been used for single-cell analyses. To prepare a platform for single-cell analysis, Yamahira et al. [7] reported a novel processing strategy for fabricating thin-bottomed round-well arrays with small dimensional errors. In this process, a mold of convex PDMS was pressed onto a polycarbonate sheet for deformation, resulting in a small dimensional error in the round-well arrays of the polycarbonate sheet over a large area without a precision mold. In this study, the platform was used for the polymerase chain reactions (PCRs) of single cells. The authors concluded that this is a promising strategy for facile, low-cost, and higher-precision microfabrication.

Microdevices are useful for point-of-care (PoC) devices, and they are utilized for the detection of chemical and biological analytes. Nguyen et al. [8] reported a summary of PoC devices for the rapid detection of coronavirus (COVID-19). This paper has been widely cited, over 260 times as of March 2022.

Finally, this editorial presents a bottom-up approach using DNAs for the fabrication of designed nanostructures. Liu et al. [9] reported a flexible DNA-ring motif with multiple joints. The shape of the DNA rings can be controlled by modulating the joints owing to the single-stranded nature of the DNAs. Additionally, the flexible DNA ring motifs can self-assemble into various structures. Although the novel motifs have not been applied to the fabrication of microdevices for biomedical analysis, they have great potential as biomedical tools based on bottom-up approaches.
